# Comparison of Chlorhexidine Gluconate, Sodium Hypochlorite, Neem Extract, and Microwave Radiation for Disinfection of Type IV Dental Stone

**DOI:** 10.1055/s-0044-1788631

**Published:** 2024-12-10

**Authors:** Bushra Jabeen, Zulfiqar A. Mirani, Muneeb A. lone, Arpit Nirkhiwale, Waqas A. Farooqui, Kashif Aslam, Muhammad Adeel Ahmed, Fahim Vohra

**Affiliations:** 1 Department of Prosthodontics, Dow International Dental College, Dow University of Health Sciences, Karachi, Pakistan; 2 Department of Microbiology, Pakistan Council of Scientific & Industrial Research Laboratories Complex, Karachi, Pakistan; 3 Department of Prosthodontics, Dr Ishrat-ul-Ebad Khan Institute of Oral Health Sciences, Dow University of Health Sciences, Karachi, Pakistan; 4 Division of Prosthodontics, Department of Restorative Dentistry, University of Minnesota, Minneapolis, Minnesota, United States; 5 Coordinator Board of Advanced Studies and Research BASR Office, Dow University of Health Sciences, Karachi, Pakistan; 6 Department of Prosthodontics, Dow Dental College, Dow University of Health Sciences, Karachi, Pakistan; 7 Department of Restorative Dental Sciences, King Faisal University, Al Ahsa, Saudi Arabia; 8 Division Of Prosthodontics, Department Of Restorative Dentistry, School of Dentistry, University of Minnesota, Minneapolis, Minnesota, United States

**Keywords:** biofilm, *Pseudomonas aeruginosa*, *Streptococcus mutans*, *Candida albicans*, disinfectants, dental materials

## Abstract

**Objective** The present study evaluated the effect of chemical disinfectants and microwave sterilization on the removal of biofilm containing *Pseudomonas aeruginosa* , *Streptococcus mutans* , and *Candida albicans* from type IV dental stone.

**Materials and Methods** One hundred twenty-eight ( *N* = 128) type IV dental cast stone specimens were prepared, and biofilms of microorganisms were cultured. Dental stone samples were subjected to disinfection protocols, including 0.5% chlorhexidine (CHX), 0.5% sodium hypochlorite (NaOCl), 20% neem extract, and microwave irradiation for 1 to 5 minutes. Colony forming unit (CFU) counts and scanning electron microscopy were utilized to witness changes in the biofilm, pre- and postdisinfection/sterilization.

**Results** For *P. aeruginosa* , significant ( *p* < 0.05) decrease in CFU counts after 1 minute (from 233 to −215) and 2, 3, and 5 minutes (from 233 to −233) were observed after CHX treatment. After microwave radiation, a significant decrease in CFU counts was also observed after 1 minute (from 233 to −130.3), 2 minutes (from 233 to −229), and 3 and 5 minutes (from 233 to −233). For *S. mutans* , a significant ( *p* < 0.05) decrease in CFU counts was observed after 1, 2, 3, and 5 minutes (from 212 to −268) after NaOCl treatment and microwave radiation (from 212 to −271 after 1 minute and from 212 to −274.3 after 2, 3, and 5 minutes). For *C. albicans* , significant ( *p* < 0.05) decrease in the CFU counts (1–5 minutes) was observed after CHX exposure, while NaOCl and microwave radiation demonstrated equal disinfection potency. Neem extract was effective to disinfect the dental stone; however, it was not as potent as the other disinfectants and microwave radiation.

**Conclusion** It was observed that exposure to CHX, NaOCl, and microwave radiation significantly reduced the microbial CFU counts. Although the use of neem extract also significantly reduced these CFU counts, this reduction was not as much as the other three tested materials. Further research exploring other chemical disinfectants with various concentrations is recommended.

## Introduction

Human saliva contains 4 million to 5 billion microorganisms/mL, while dental plaque contains 10 to 1,000 billion microorganisms/g. 1 Dental plaque is a biofilm that contains a combination of microorganisms and exopolysaccharides, and its presence can induce various oral diseases including periodontitis. 2 Gypsum-based products are widely used in dentistry to fabricate casts after taking dental impressions. 3 Dental gypsum products can range from an exceedingly soft impression plaster (type I) to extremely strong, wear-resistant, low expansion, and high strength containing stone (type IV). 4 Master casts for the fabrication of indirect restorations are generally made from the hardest stone type (type IV). 5 These stone casts contain a high risk of cross-contamination as various infectious microbiological agents could be transferred from the patient's saliva, blood, and plaque onto these casts. 6 These infected casts not only pose threat to the dentist and assistant handling them but also to the technicians in the laboratory facilities. 7

Disinfection is the process of completely eliminating all vegetative forms of microorganisms from inanimate things, except bacterial spores. A previously recommended procedure to disinfect dental impressions was to wash them under running tap water. However, this technique only eliminated 40% of the microorganisms, resulting in inefficient disinfection. 7 Commonly used disinfectants for dental impression materials include iodophor, glutaraldehyde, sodium hypochlorite (NaOCl), benzalkonium chloride, isopropyl and ethyl alcohol, chlorhexidine (CHX), and ozone water. 7 In the literature, many methods have been mentioned for the disinfection of dental casts using these disinfectants. A few of these methods include spraying disinfection solutions over the cast, immersing the cast into the disinfectants, and integrating disinfectants inside the cast. 8 The main drawbacks of the first two methods are the decrease in surface quality and the inability of surface disinfectant to penetrate the cast, which might lead to contamination of the cast's interior. 9 Concerning the incorporation of disinfectants in the water while preparing the cast, the literature shows that it can negatively affect the cast's mechanical properties. 9 Therefore, the quest to find a technique that results in optimum disinfection with minimal effect on dental stone cast properties continues.

Sterilization is the process of completely eliminating or destroying all microbial life (including vegetative and spore forms), and it can be accomplished using various physical and chemical techniques. 10 Microwave sterilization of the dental stone cast is an effective method to eliminate microorganisms and their spores. A previous study by Campanha et al demonstrated that exposing bacterial suspensions to microwave radiation decreased the number of viable cells and encouraged the leaching of DNA and protein from microorganisms. 11 These findings imply that microwave irradiation may impact cell membrane permeability, integrity, and metabolism, which could result in cell death.

Effective disinfection is required due to the high infection risks posed by the presence of biofilms on dental materials. This disinfection efficacy of microwave sterilization, disinfectants, CHX, and natural alternatives varies. Hence, the rationale of the present study was to compare the efficacy of different chemical disinfectants and microwave sterilization on biofilm removal from type IV dental cast stone. The null hypothesis (H _o_ ) of the present study was that chemical disinfectants and microwave sterilization would demonstrate similar efficacy in removing biofilm from dental cast stone.

## Materials and Methods

The project was reviewed and approved by the Institutional Review Board (IRB) committee of Dow University of Health Sciences (IRB-1604/ DUHS/approval/2020).

### Preparation of Dental Cast Stone Specimens

One hundred twenty-eight ( *N* = 128) dental cast stone specimens measuring 30 × 5 × 5 mm (W × L × H) were prepared, following the recommendations of a previous study. 12 Briefly, 100 g of type IV dental stone powder (GC Fujirock, GC America, Illinois, United States) was weighed to the nearest ± 0.1 g using a physical balance. This powder was then transferred to a clean silicon rubber bowl, and recommended amount of distilled water was added to it according to the manufacturer's instruction and mixed manually by using a stainless steel spatula until a smooth consistency was achieved. Then, the silicone bowl was placed over the vibrator for 30 seconds to reduce porosity and to avoid the creation of air bubbles in the poured stone. The mixed dental stone was then poured into the stainless steel mold and leveled by using a glass slab to get a smooth and consistent surface. The cast stone was allowed to be set for an hour at room temperature (24–25°C). After setting the type IV dental stone specimen, the mold was opened, and the stone was removed from the mold and left to air dry for 2 hours at room temperature.

### Cultivation of Microorganisms for Biofilm

A single colony of bacteria, that is, *Pseudomonas aeruginosa* and *Streptococcus mutans* were inoculated into 10 mL Tryptic Soy Broth (TSB; Oxoid, Basingstoke, Hampshire, UK) and incubated at 35°C for 1 day. After 24 hours, the loop full from TSB was streaked on Tryptic Soy Agar (TSA) and incubated for 24 hours at 35°C. The isolated bacterial colonies from the TSA were then picked for Gram staining to confirm the purity of the bacteria. A single colony of the provided culture of *Candida albicans* was inoculated in TSB and incubated at 35°C for 48 hours. Then, a loop full from TSB was streaked on Dichloran Rose-Bengal Chloramphenicol (DRBC) agar plate and incubated at 35°C for 48 hours. A single fungal colony was picked from the DRBC agar, and simple staining was performed to check the purity of this culture. The bacterial and fungal load was adjusted according to 0.5 McFarland turbidity standards (1.5 × 10 ^8^ ) to conduct further experiments in this study. 13 14

### Biofilm Formation Assay, Grouping, and Microbial Count Check

For this part of the study, all the dental cast stone specimens were submerged into Brain Heart Infusion broth (Difco, Detroit, Michigan, United States) containing 0.5 mL of overnight culture of *P. aeruginosa* , *S. mutans* , and *C. albicans* . These cast stone specimens were incubated for 48 hours at 35°C. Following incubation, the specimens were removed and cleaned with distilled water to remove the dirt and cells that were weakly adhered. The samples were vortexed for 2 minutes in phosphate-buffered saline (pH: 7). Following vortexing, the specimens with biofilm were checked for the microbial count (colony forming units [CFUs]). These samples were then divided equally and randomly into four groups (Gps) and subjected to four treatment protocols ( *n* = 32/treatment Gp) for 1, 2, 3, and 5 minutes ( *n* = 8/1 minute, *n* = 8/2 minutes, *n* = 8/3 minutes, and *n* = 8/5 minutes); Gp-A: the samples were exposed to a 100-mL solution inside a glass beaker containing chemical disinfectant and distilled water in 1:10 ratio (10 mL of 0.5% CHX in 90 mL of distilled water), Gp-B: the samples were exposed to a 100-mL solution inside a glass beaker containing chemical disinfectant and distilled water in 1:10 ratio (10 mL of 0.5% NaOCl in 90 mL of distilled water), Gp-C: the samples were exposed to a 100-mL solution inside a glass beaker containing chemical disinfectant and distilled water in 1:10 ratio (10 mL of 20% neem extract in 90 mL of distilled water), and Gp-D: the samples were sterilized using microwave radiation at 450 W by first placing them inside a glass beaker containing 100 mL of distilled water and then placing the beaker inside a microwave oven (Samsung 2,450 MHz, 800 W).

After the above-mentioned treatments, the specimens were then emulsified in 10 mL of sodium phosphate buffer solution for 5 minutes. The plate count was checked by subculturing 1 mL of buffer on a plate count agar, incubated for 24 hours at 35°C, after that the microbial count ( CFUs) was checked again.

### Scanning Electron Microscopy

Scanning electron microscopy (SEM) was used to study biofilm formation on type IV dental cast stone. After biofilm development, the stone cast was washed with deionized water to remove debris and loosely attached cells from the consortia. After washing, the biofilm was scraped from the type IV stone and fixed on a 4-mm glass slide by 15 seconds exposure to microwave radiation (450 W). The biofilm was negatively stained with 0.2% uranyl acetate for 30 seconds. After staining, the slides were then observed under an SEM (JOEL-JSM IT-100, Tokyo, Japan). SEM was repeated again to witness changes in the biofilm, pre- and postdisinfection/sterilization.

### Statistical Analysis

Mean and standard deviation of quantitative variables were reported, and data analysis was performed using IBM SPSS statistics software version 21 (Inc., United States). For CFU counts, Mann–Whitney's test was applied as the data were not normally distributed. A *p* -value < 0.05 or less was considered significant.

## Results

### CFU Counts

**Fig. 1:**
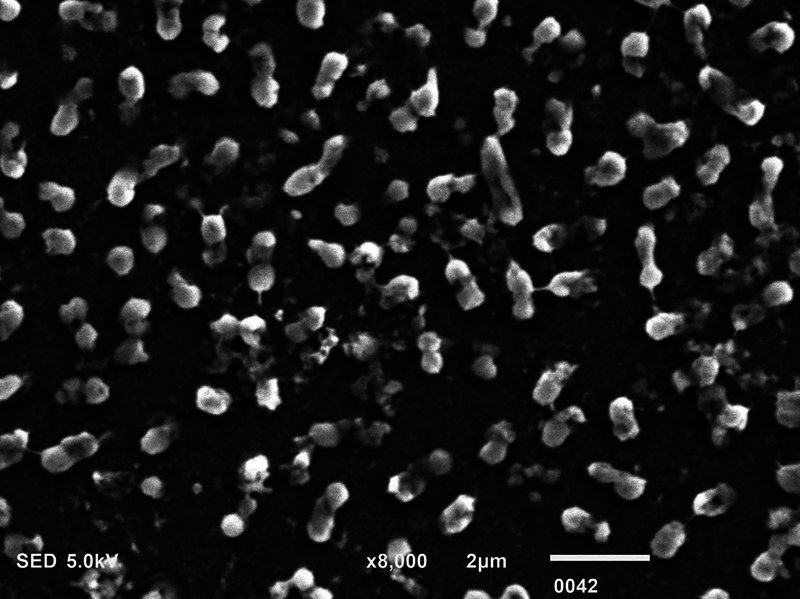
Scanning electron microscopy micrograph showing dead *Pseudomonas aeruginosa* postmicrowave sterilization (magnification ×8,000).

The contaminated type IV stone specimen was found to carry 233, 212, and 268 CFU counts of *P. aeruginosa* , *S. mutans* , and *C. albicans* on its surface after 48-hour exposure. For *P. aeruginosa* , after the exposure to different chemical disinfectants and microwave sterilization, it was noticed that the CFU counts significantly ( *p* < 0.05) dropped after 1, 2, 3, and 5 minutes for all the treatment groups, compared with the controls ( Table 1 ) ( Fig. 1 ). After the CHX (Gp-A) exposure, the CFU counts decreased to −215 after 1 minute and then reduced to −233 after 2, 3, and 5 minutes. After the exposure to NaOCl (Gp-B), the counts decreased to 183.3 after 1 minute, then to −225.33 after 2 minutes, and then were recorded as −232.6 after 3 and 5 minutes. For neem extract (Gp-C), the counts were decreased to −17 after 1 minute and −34 after 5 minutes (counts were not recorded for 2 and 3 minutes). For microwave sterilization, the counts decreased to 130.3 after 1 minute, −229 after 2 minutes, and then to −233 after 3 and 5 minutes ( Table 1 ).

**Table 1:** CFU count comparison for *Pseudomonas aeruginosa*

Exposure time	Chlorhexidine 0.5%	Sodium hypochlorite 0.5%	Neem extract 20%	Microwave sterilization
1 min	−215 (<0.001) a	183.3 (<0.001) a	−17 (<0.001) a	−130.3 (<0.001) a
2 min	−233 (<0.001) a	−225.33 (<0.001) a	–	−229 (<0.001) a
3 min	−233 (<0.001) a	−232.6 (<0.001) a	–	−233 (<0.001) a
5 min	−233 (<0.001) a	−232.6 (<0.001) a	−34 (<0.001) a	−233 (<0.001) a

Abbreviation: CFU, colony forming unit.

a Significant at *p* < 0.05 versus the control (control CFU count = 233).

**Fig. 2:**
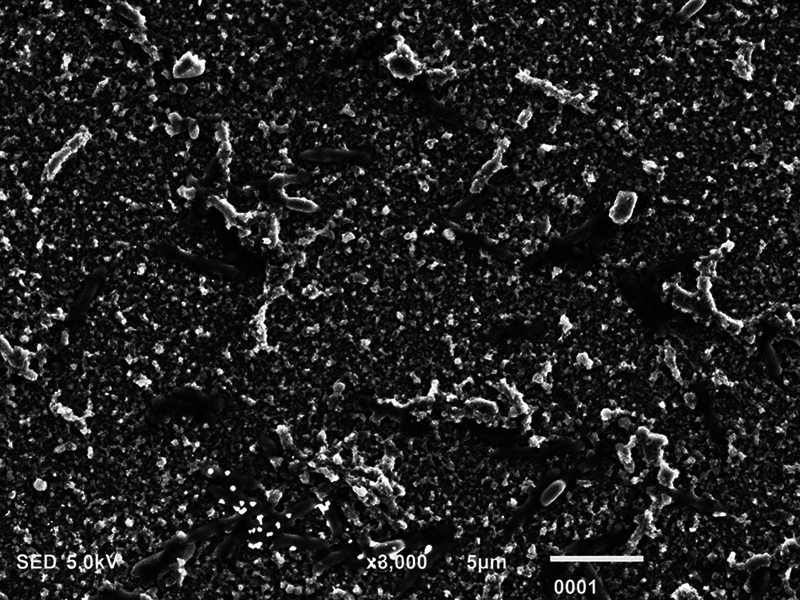
Scanning electron microscopy micrograph showing dead *Streptococcus mutans* postmicrowave sterilization (magnification ×3,000).

For *S. mutans* , after the exposure to different chemical disinfectants and microwave sterilization ( Fig. 2 ), it was noticed that the CFU counts significantly ( *p* < 0.05) dropped after 1, 2, 3, and 5 minutes for all the treatment groups (except neem extract after 1 minute exposure), compared with the controls ( Table 2 ). After the CHX (Gp-A) exposure, the CFU counts decreased to −268 after 1 minute, and then the same count (−268) was noticed at 2, 3, and 5 minutes. After the exposure to NaOCl (Gp-B), the counts decreased to −274.3 after 1 minute, and then the exact count (−274.3) was recorded at 2, 3, and 5 minutes. For neem extract (Gp-C), the counts were decreased to −2.33 after 1 minute (nonsignificant) and −6 after 5 minutes (counts were not recorded for 2 and 3 minutes). For microwave sterilization, the counts decreased to −271 after 1 minute and −274.3 after 2 minutes, and the exact count was recorded at 3 and 5 minutes ( Table 2 ).

**Table 2:** CFU count comparison for *Streptococcus mutans*

Exposure time	Chlorhexidine 0.5%	Sodium hypochlorite 0.5%	Neem extract 20%	Microwave sterilization
1 min	−268 (<0.001) a	−274.3 (<0.001) a	−2.33 (<0.351)	−271 (<0.001) a
2 min	−268 (<0.001) a	−274.3 (<0.001) a	–	−274.3 (<0.001) a
3 min	−268 (<0.001) a	−274.3 (<0.001) a	–	−274.3 (<0.001) a
5 min	−268 (<0.001) a	−274.3 (<0.001) a	−6 (<0.026) a	−274.3 (<0.001) a

Abbreviation: CFU, colony forming unit.

a Significant at *p* < 0.05 (control CFU count = 212).

**Fig. 3:**
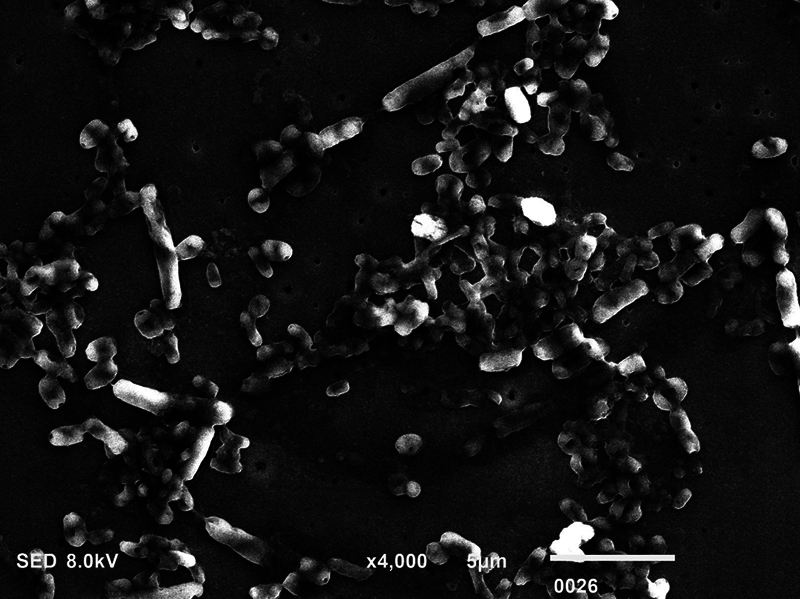
Scanning electron microscopy micrograph showing dead *Candida albicans* postmicrowave sterilization (magnification ×4,000).

For *C. albicans* , after the exposure to different chemical disinfectants and microwave sterilization ( Fig. 3 ), it was noticed that the CFU counts significantly ( *p* < 0.05) dropped after 1, 2, 3, and 5 minutes for all the treatment groups, compared with the controls ( Table 3 ). After the CHX (Gp-A) exposure, the CFU counts decreased to −215 after 1 minute, to −233 after 2 minutes, and then the same count (−233) was noticed after 3 and 5 minutes. After the exposure to NaOCl (Gp-B), the counts decreased to −170 after 1 minute, to −196 after 2 minutes, and then to −211 after 3 and 5 minutes. For neem extract (Gp-C), the counts were decreased to −68 after 1 minute, and then the same count (−68) was observed after 5 minutes (counts were not recorded for 2 and 3 minutes). For microwave sterilization, the counts decreased to −187.3 after 1 minute, −199 after 2 minutes, and −211 after 3 and 5 minutes ( Table 3 ).

**Table 3:** CFU count comparison for *Candida albicans*

Exposure time	Chlorhexidine 0.5%	Sodium hypochlorite 0.5%	Neem extract 20%	Microwave sterilization
1 min	−215 (<0.001) a	−170 (<0.001) a	−68 (<0.001)	−187.3 (<0.001) a
2 min	−233 (<0.001) a	−196 (<0.001) a	–	−199 (<0.001) a
3 min	−233 (<0.001) a	−211 (<0.001) a	–	−211 (<0.001) a
5 min	−233 (<0.001) a	−211 (<0.001) a	−68 (<0.001) a	−211 (<0.001) a

Abbreviation: CFU, colony forming unit.

a Significant at *p* < 0.05 (control CFU count = 268).

### SEM Analysis

**Fig. 4:**
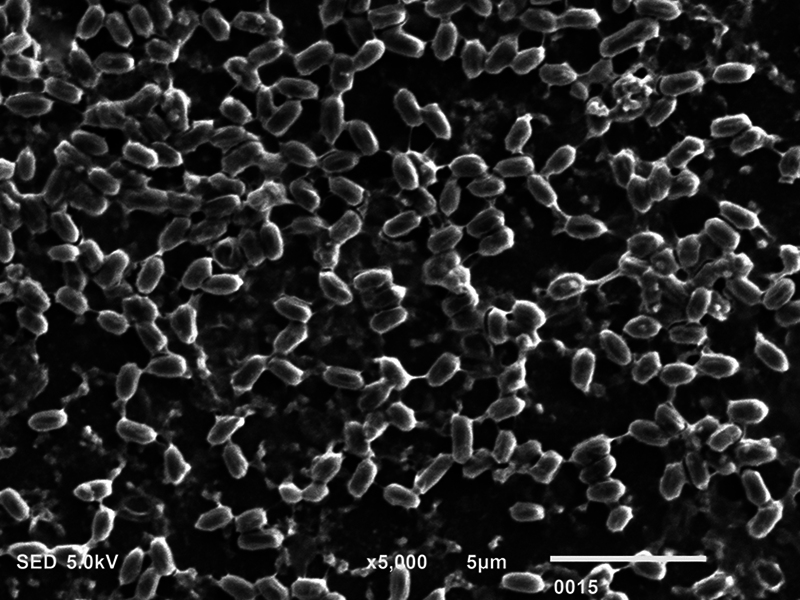
Scanning electron microscopy micrograph showing live *Pseudomonas aeruginosa* premicrowave sterilization (magnification ×5,000).

The biofilm consortia of *P. aeruginosa* , *S. mutans* , and *C. albicans* demonstrated that it harbored cells of these microorganisms, which were aggregated, and surrounded with extracellular matrix (ECM) material ( Figs. 4 5 6 premicrowave sterilization). After disinfection and microwave sterilization, dead cells were found to adhere to the surface as a tiny, agglomerated mass of irregularly shaped cells ( Figs. 1 2 3 postmicrowave sterilization).

**Fig. 5:**
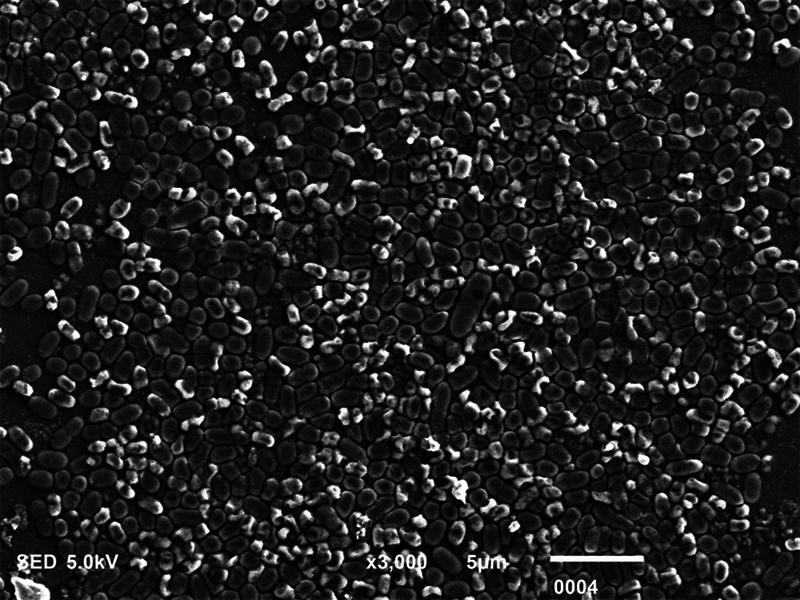
Scanning electron microscopy micrograph showing live *Streptococcus mutans* premicrowave sterilization (magnification. ×3,000).

**Fig. 6:**
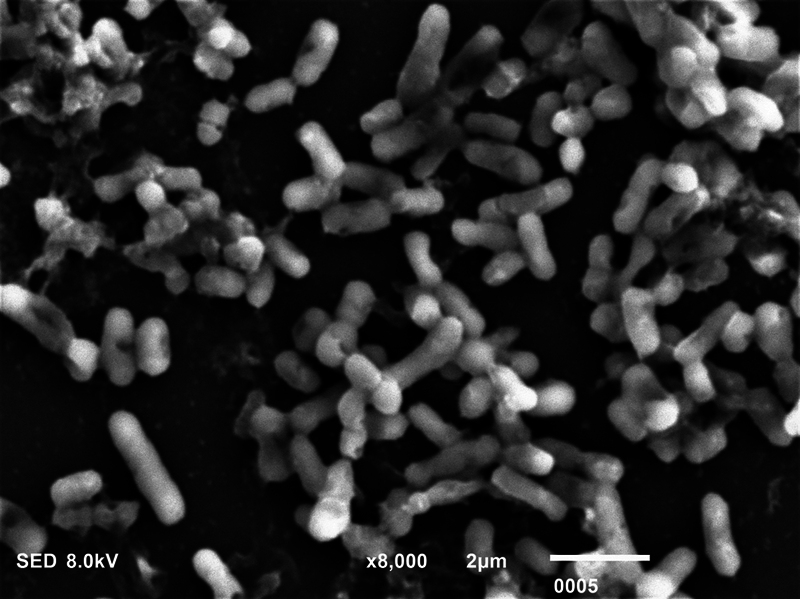
Scanning electron microscopy micrograph showing live *Candida albicans* premicrowave sterilization (magnification ×8,000).

## Discussion

Based on the findings of this study, the H _o_ that chemical disinfectants, natural compound, and microwave radiation are equally effective in disinfecting type IV stone cast was rejected. It was observed that various chemical disinfectants, neem extract, and microwave sterilization procedure demonstrated varying efficacy in reducing *P. aeruginosa* , *S. mutans* , and *C. albicans* CFU counts. The requirement for disinfection is further emphasized by the prospect that a dental prosthesis might be a source of infection and cross-contamination between patients and dental staff. 15 Therefore, dental appliance disinfection poses a significant problem for clinicians, assistants, and laboratory workers and requires urgent resolution.

Concerning *P. aeruginosa* , it was noticed that the highest decrease in the CFU counts after 1 and 2 minutes were noticed after the exposure to CHX, while after 3 and 5 minutes, CHX and microwave sterilization were equally effective. CHX is an antimicrobial agent, which, in low concentration, is bacteriostatic, while in high concentration, it is bactericidal. 16 CHX molecule is cationic, so it is attracted to the negatively charged bacterial surfaces. 17 After a bond is formed between the CHX molecule and negatively charged surfaces of the bacteria in the biofilm, particular and robust adsorption to phosphate-containing components that make up the bacterial cell's surface occurs. 18 Passive diffusion causes penetration through the bacterial cell wall by drawing substances to the cell's cytoplasmic membrane, where they harm and compromise the integrity of the bacterial cytoplasmic membrane. 19 Most oral surfaces, such as mucous membranes, teeth, and salivary glycoproteins, have a negative charge, making cationic CHX molecules cling to these surfaces and preventing bacterial adhesion, allowing substantivity for ∼12 hours. 20 It is anticipated that this mode of action of CHX must have played a crucial and effective role in reducing the count of *P. aeruginosa* . Microwave sterilization was found to be equally effective as CHX exposure after 3 and 5 minutes. Our results agree with the previous findings of Anaraki et al who demonstrated that microwave sterilization effectively reduces microbial load (including microorganisms such as *P. aeruginosa* , *S. mutans* , and *C. albicans* ) from the dental cast stones. 21 Few other previous studies have conveyed the same findings and verified that microwave sterilization is an effective method to disinfect dental cast stones. 22 23 During microwave sterilization, magnetrons produce microwaves and propagate in a strong line along the waveguide. When this radiation is absorbed by materials that contain water, the water molecules rub against one another in an alternating electrical field. The heat created by this conversion of energy is thought to quickly kill microorganisms with a high water content. 24 It is predicted that this mechanism of action of microwave sterilization was involved in effective removal of microorganisms from the biofilm and reduction of their CFU counts. In the present study, NaOCl disinfection also reduced the CFU counts for *P. aeruginosa* . Previously, Choudhury et al reported that disinfection of dental impressions with 0.5% NaOCl results in 99% reduction of the counts of *P. aeruginosa* in the first 10 minutes of exposure, 25 and our results are in agreement with this study. We also noticed that the reduction of CFU counts by NaOCl was less than the microwave irradiation. A previous study also reported similar findings and demonstrated that for the removal of *P. aeruginosa* from dental cast stones, microwave irradiation is more potent than the use of NaOCl. 26 The variations in the microbial load for casts treated with NaOCl might be the result of either the ineffectiveness of dipping the casts in 0.5% NaOCl for 1, 2, 3, and 5 minutes or the inability of this concentration to eradicate all the bacteria. 26 Still, this needs further investigation.

Regarding *S. mutans* , it was noticed that the highest decrease in the CFU counts was observed after microwave sterilization (mechanism explained above) and exposure to NaOCl equally, while exposure to neem extract was comparatively less effective. A previous study has shown that exposure to NaOCl effectively removes dental plaque bacteria and significantly reduces the CFU counts of *S. mutans* . 27 Our findings agree with this study as we also observed a significant reduction in CFU counts of *S. mutans* immediately after exposure to 0.5% NaOCl. The NaOCl solution, also known as bleach, is composed of sodium cation and hypochlorite anion. NaOCl kills microorganisms by various methods, including oxidation, protein denaturation, and damaging their lipids and DNA, 28 and these mechanisms could be involved in the reduction of *S. mutans* CFU counts in the present study. It should be noted although NaOCl effectively kills various viruses, fungi, and bacteria, a concentration of more than 0.5% is not recommended as it could change the physical properties of the cast stone. 7 This was the reason that we did not use a high concentration NaOCl solution for our study.

For *C. albicans* , the highest reduction in the CFU counts was demonstrated by CHX, followed closely by microwave sterilization and NaOCl exposure. A previous study has demonstrated similar findings and reported that microwave irradiation of dental cast stone reduces the microbial counts of *C. albicans* (and other microorganisms), and the efficacy is comparable to a conventional chemical disinfectant (NaOCl). 29 One difference between this previous study 29 and our study is the energy level of the microwave irradiation. Although our study used less energy (450 W) compared with their study (600 W), we observed better disinfection potential associated with microwave irradiation, compared with NaOCl use. Less energy usage is more environmental friendly and could result in reduction of financial costs for dental practices. The neem extract was the least effective disinfectant for *C. albicans* in this study. *Azadirachta indica* , also called neem tree, has been used to treat skin diseases, dental disorders, inflammation, and infections. 30 Neem has shown its efficacy against various dental pathogens, including *S. mutans* , *S. aureus* , and *C. albicans* . 31 Neem extract is composed of multiple compounds that have antibacterial and antifungal properties. Azadirachtin, an active neem compound, exhibits broad-spectrum antimicrobial activity, while limonoids (such as nimbin and nimbidin) have potent antibacterial and antifungal properties. 32 33 While it cannot be absolutely established, it is anticipated that these active compounds must have played in role in the decrease of CFU counts of microorganisms assessed in this study, although it was less than all other disinfection/sterilization methods.

On SEM analysis, it was observed that biofilm consortia of *P. aeruginosa* , *S. mutans* , and *C. albicans* changed from aggregated cells with ECM to having dead cells on the surface after disinfection and microwave sterilization. The same findings were conveyed before by Choudhry et al, who also demonstrated that biofilm consortia change from having live to dead cells after various disinfection procedures and upon exposure to microwave irradiation, 15 and our findings are in line with their study. The mechanism via which each disinfectant and microwave sterilization work to reduce the CFU counts and thus impact biofilm consortia being visualized via SEM has been explained in detail previously in this section.

Based on the findings of the present study, the following clinical recommendations could be made:

1. Dental practices should consider incorporating CHX or NaOCl for routine chemical disinfection of dental cast stones.
2. For rapid and efficient sterilization, microwave irradiation is highly recommended.
3. While neem extract is effective in disinfecting dental cast stones, its potency is comparatively lower than CHX, NaOCl, and microwave radiation. Neem extract may be considered as an alternative disinfectant, but further research is needed to optimize its use.

A major strength of the present study includes the testing of four materials in reducing the CFU counts of different microorganisms from the surface of dental cast stone at various time points. Among the limitations, a major limitation of the present study includes its *in vitro* nature. It should be noted that the current study results could be different if this study is conducted in an *in vivo* environment due to its dynamic, ever-changing nature. Another limitation was the use of specific concentrations and exposure times of chemical disinfectants. The readers should be aware that increasing or decreasing the concentration and exposure time of these chemical disinfectants could yield different results. Future studies aimed at testing other materials with various concentrations and time points are recommended.

## Conclusion

The treatment of dental cast stones with CHX, NaOCl, and microwave radiation significantly reduced the CFU counts of mircoorganisms in this study. Although the use of neem extract also significantly reduced these CFU counts, this reduction was not as much as the other three tested materials. Further research exploring other chemical disinfectants with various concentrations is recommended.
